# De Novo Whole Genome Assemblies of Unusual Case‐Making Caddisflies (Trichoptera) Highlight Genomic Convergence in the Composition of the Major Silk Gene (*h‐fibroin*)

**DOI:** 10.1002/jez.b.23301

**Published:** 2025-05-19

**Authors:** Xiling Deng, Steffen U. Pauls, Ryoichi B. Kuranishi, Paul B. Frandsen, Jacqueline Heckenhauer

**Affiliations:** ^1^ Senckenberg Research Institute and Natural History Museum, Terrestrial Zoology Frankfurt am Main Germany; ^2^ LOEWE Centre for Translational Biodiversity Genomics (LOEWE‐TBG) Frankfurt am Main Germany; ^3^ Institute for Insect Biotechnology Justus‐Liebig‐University Gießen Germany; ^4^ Graduate School of Science Chiba University Chiba Japan; ^5^ Kanagawa Institute of Technology Atsugi Kanagawa Japan; ^6^ Department of Plant & Wildlife Sciences Brigham Young University Provo Utah USA; ^7^ Data Science Lab, Office of the Chief Information Officer Smithsonian Institution Washington District of Columbia USA

**Keywords:** aquatic insects, evolutionary innovation, long‐read sequencing, underwater silk

## Abstract

Trichoptera (caddisflies) is one of the most species‐rich orders of aquatic insects. Species of caddisflies cover a broad ecological diversity as exemplified by various uses of underwater silk secretions. Diversity of silk use generally aligns with the evolution of major caddisfly lineages, specifically at the subordinal level: Annulipalpia (retreat makers) and Integripalpia (cocoon and tube‐case makers). However, silk use within suborders differs for a few exceptional species in these clades. In this study, we provide the first whole genome assemblies and annotations for two unusual Integripalpia species: *Limnocentropus insolitus*, whose hard tube‐case is anchored to boulders by a rigid, elongated silken stalk, and *Phryganopsyche brunnea* which builds a “floppy” cylindrical case that lacks the typical robustness of tube‐cases. Its texture rather resembles that of the flexible retreats built by Annulipalpia. Using the two high‐quality genome assemblies, we identified and annotated the major silk gene, *h‐fibroin*, and compared its amino acid composition across various groups, including retreat, cocoon, and tube‐case makers. Our phylogenetic analysis confirmed the phylogenetic position of the two species in the tube‐case‐making clade. The major silk gene of *L. insolitus* shows a similar amino acid composition to other tube‐case‐making species. In contrast, the amino acid composition of *P. brunnea* resembles that of retreat‐making species, in particular with regard to the high content of proline. This is consistent with the hypothesis that proline could be linked to enhanced extensibility of silk fibers. Taken together, our results underscore the role of silk genes in shaping the evolutionary ecology of retreat‐ and tube‐case‐making in caddisflies.

## Graphical abstract

The major silk gene, *h‐fibroin*, was annotated in high‐quality genomes of two unusual case‐making Trichoptera. H‐Fibroin of *Limnocentropus insolitus* shows a similar amino acid composition to other case‐making species, while the amino acid composition of h‐fibroin of *Phryganopsyche brunnea* resembles that of retreat‐making species. This underscores the role of silk genes in shaping the evolutionary ecology of retreat‐ and tube‐case‐making in Trichoptera.

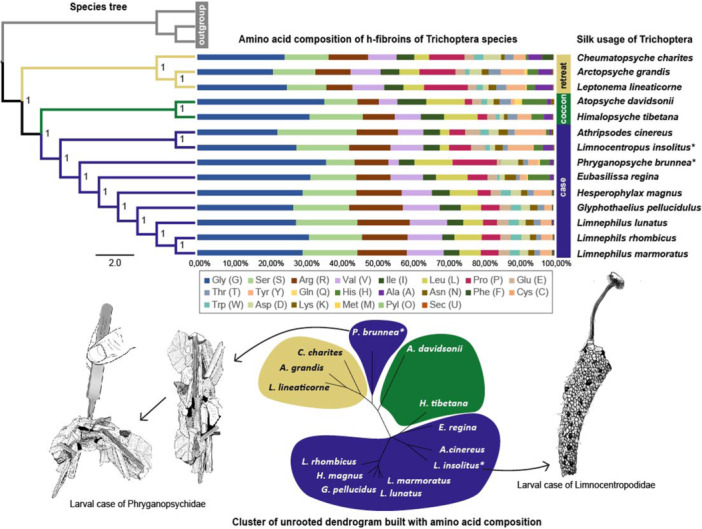

## 1 Introduction

Silk is used by many insect species for various purposes, including shelter, protection and predation (Craig et al. 1999; Sutherland et al. 2010). The diversity of silk across insect species arises from the unique properties of proteins, which are determined by their molecular sequences. With recent advances in sequencing technology, the improved characterization of silk‐associated genes enables exploring how the molecular sequence of these genes might be linked to silk properties.

In the insect superorder Amphiesmenoptera, which contains the orders Lepidoptera (moths and butterflies) and Trichoptera (caddisflies), the *heavy‐chain fibroin* (*h‐fibroin*) gene encodes the primary protein component of silk (Jin and Kaplan 2003). In most Lepidoptera, the *h‐fibroin* is associated with both light chain fibroin (*l‐fibroin*) and P25, whereas in Trichoptera, it is only associated with *l‐fibroin* (Sutherland et al. 2010; Yonemura et al. 2009). Based on previous studies, the *h‐fibroin* gene sequences are remarkably long (> 20,000 bp) and are characterized by a highly repetitive region embedded between conserved termini (e.g., Fedič et al. 2003; Frandsen et al. 2019; Mita et al. 1994). The length of the gene, as well as its internal repetitive region, posed challenges for sequencing and assembling the complete gene, especially when only short‐read sequencing techniques were available. However, advanced, accurate long‐read sequencing techniques (e.g., PacBio HiFi) have enabled the generation of high‐quality, full‐length *h‐fibroin* sequences (as shown in Hotaling et al. 2023; Heckenhauer et al. 2023; Kawahara et al. 2022). Comparison of these full‐length h‐fibroins revealed variations in the sequences of repeat modules and amino acid composition across clades of caddisflies with different silk use behaviors. Previous studies suggest that these differences may be associated with different properties of the silk (Frandsen et al. 2019; Heckenhauer et al. 2023). In addition, PacBio long‐reads can be assembled into haploid‐resolved genomes using new assembly tools (e.g., Hifiasm, Cheng et al. 2021, 2022). This approach was successfully used to recover full‐length, fully resolved sequences for both alleles of h‐fibroin, unveiling substantial and previously hidden heterozygosity within the individual (Frandsen et al. 2023).

In contrast to the majority of Lepidoptera species, nearly all Trichoptera species have aquatic larval and pupal stages. During these life stages, caddisflies use silk to construct diverse underwater structures that facilitate adaptation to their specific habitats (Frandsen et al. 2024). Trichoptera are divided into two suborders, Annulipalpia and Integripalpia, based on the morphology of the adult mouthpart maxillary palp segments (Ross 1967). This classification is supported by molecular evidence (Frandsen et al. 2024; Thomas et al. 2020). Species from the suborder Annulipalpia are referred to as net‐ and fixed‐retreat makers because their larvae build stationary shelters with silk and other material in moving water (Morse et al. 2019). These stationary shelters are used as physical protection against predators and facilitate the collection of food. Some species of the suborder Integripalpia are free‐living until pre‐pupation. These species are often referred to as cocoon makers because they create closed, semi‐permeable pupal cocoons that maintain a controlled osmotic environment and are fixed to the substrate under a dome of pebbles (Wiggins 2004). However, most members of the suborder Integripalpia build portable tube‐cases that are lined with silk, allowing them to exploit a wide range of aquatic environments, encompassing both lotic and lentic habitats (Wiggins 2004). This group is referred to as the Phryganides. Their tube‐cases vary in shape (conical, spiral, flattened) and are made of various materials (sand grains, rock fragments, mollusc shells, or plant material) glued together by silk. For pupation, most of the species simply seal off the tube‐case openings with silk to construct their pupal chamber. The tube‐case architectures are generally conserved at the genus and even family level, and are closely linked to feeding, defense, and other behaviors within specific habitats (Wiggins 2004). However, in some lineages, the tube‐case architecture is distinct among close relatives but similar among more distantly related groups. For instance, species of *Phryganopsyche*, the sole genus in the family Phryganopsychidae, construct remarkably unique cases compared to other tube‐case makers: these cases are significantly longer than the larvae and bend under their own weight when removed from the water, thus somewhat resembling an Annulipalpian retreat in structure. However, in contrast to Annulipalpian retreats, these “floppy” cases are not attached to the substrate and remain portable (Wiggins 2004). Another example is *Limnocentropus*, the only genus in the family Limnocentropodidae. Species in this group produce tube‐cases of rock fragments with silken denticles, which are anchored to boulders in turbulent streams by a robust, elongated silken stalk. Unlike the portable cases produced by other tube‐case‐making caddisflies, the tube‐cases of *Limnocentropus* are attached to the substrate, similar to retreat‐making species. In this study, we provide the first whole‐genome assemblies and annotations for the caddisfly families Phryganopsychidae (*Phryganopsyche brunnea*) and Limnocentropodidae (*Limnocentropus insolitus*). We used these genomes to compare the primary structure and amino acid composition of full‐length sequences of h‐fibroin with previously published h‐fibroin sequences of caddisflies. In addition, phylogenetic reconstructions based on the conserved N‐terminal region of the h‐fibroin, as well as Benchmarking Universal Single‐Copy Orthologue (BUSCO) genes, supported the classification of *L. insolutus* and *P. brunnea* as tube‐case makers (Phryganides). Furthermore, we propose that the “floppy” cases of *P. brunnea* may have mechanical properties similar to those of retreat maker species, potentially due to alterations in the sequence of h‐fibroin.

## 2 Materials and Methods

### 2.1 Sampling and Sequencing

In this study, we collected a female larva of *L. insolitus* in Fukushima Prefecture, Japan (N 37°34′46.63″ E 140°19′28.34″, elevation ~636 m a.s.l.). We used the whole body of this specimen for DNA extraction. For *P. brunnea*, we collected a female adult at a different site from the same prefecture (N 37°36′6.65″ E 140°12′36.63″, elevation ~750 m a.s.l.) and extracted DNA from the whole specimen. For sex determination, see Supporting Information S1: Tables S1 and S2.

The DNA extraction and library preparation were performed following Kawahara et al. (2022). The libraries were barcoded and sequenced together on a single PacBio Revio SMRT cell at the Brigham Young University DNA Sequencing Center. By producing long, high‐fidelity sequence reads, this sequencing approach provides haplotype phasing from a single library and sequencing run.

### 2.2 Genome Size Estimation, Genome Assembly, and Quality Control

We used a *k‐mer* counting approach to estimate the genome size of the two genomes with Jellyfish v.2.3.0 (Marçais and Kingsford 2011) and GenomeScope v.2.0 (Ranallo‐Benavidez et al. 2020; Vurture et al. 2017). After tallying all 21‐*mers* with Jellyfish count with the parameters recommended by Genomescope2 [canonical “k‐mers” (‐C), initial hash size (‐s) 1000000000], we exported a histogram of *k‐mer* occurrences using the Jellyfish histo function. We then used the histogram to generate genome *k‐mer* profiles using the GenomeScope 2.0 online web tool by setting the *k‐mer* length as 21 and ploidy as 2.

We assembled the PacBio HiFi reads of the two genomes into contigs using Hifiasm v.0.18.7 (Cheng et al. 2021, 2022). In contrast to previously developed programs that either collapse heterozygous alleles into one consensus copy or fail to cleanly separate the haplotypes to produce high‐quality phased assemblies, the algorithm of Hifiasm represents the haplotype information in a phased assembly graph and thus produces a primary and alternate assembly (Cheng et al. 2021, 2022).

We detected and filtered out potential contamination of the two primary genome assemblies as follows: We first mapped the raw reads back to the assemblies using minimap2 v.2.12 (Li 2018) with the parameter for PacBio HiFi/CCS genomic reads (‐ax asm20), and sorted the output bam files with SAMtools v.1.17 (Danecek et al. 2021). We then used Megablast (Morgulis et al. 2008) to search the assemblies against the NCBI nucleotide sequence database (nt.gz) with entries from all traditional divisions of GenBank, EMBL, and DDBJ (available from ftp://ftp.ncbi.nlm.nih.gov/blast/db/) with expectation value threshold (‐evalue 1e−25) and to return the hits in a custom output format (‐outfmt “6 qseqid staxids bitscore std”). Subsequently, we created a BlobTools database with the assembly file, blast output, and the indexed bam file from the mapping and plotted the results using BlobTools (Laetsch and Blaxter 2017). We then identified mitochondrial contigs using MitoHiFi v.3.2 with an invertebrate mitochondrial code setting (‐o 5, Uliano‐Silva et al. 2023) using the mitogenome of *Limnocentropus hysbald* (GenBank accession number: OL678029.1) as a reference mitogenome for *L. insolitus* and the mitogenome of *Phryganopsyche latipennis* (GenBank accession number: KX385012.1) for *P. brunnea*. We then removed the contigs detected by MitoHiFi from the assemblies using SAMtools. Mitogenomes are available at figshare: https://doi.org/10.6084/m9.figshare.28645466.v1. Finally, we filtered out two contigs from the genome assembly of *L. insolitus* reported as contamination by NCBI during the data upload process (ptg000032l: length 1,373,614 bp, a‐proteobacteria; contig ptg000230l: length 16,064 bp, mycoplasma) by the Foreign Contamination Screen tool (FCS, Astashyn et al. 2024) for GenBank submissions.

We examined the quality of the two contamination‐free, nuclear genome assemblies using QUAST v.5.0.2 (Mikheenko et al. 2018) and BUSCO v.5.4.3 (Manni et al. 2021) with the obd10 Endopterygota reference gene set. We used BlobToolKit (Challis et al. 2020) to visualize the results in a snail plot. We additionally estimated the back‐mapping rates of the HiFi reads to the assemblies using minimap2 and BamQC (Qualimap v.2.2.1, Okonechnikov et al. 2016).

### 2.3 Repeat and Gene Annotation

We identified and classified the repetitive elements de novo by first creating a library of consensus repeat sequences using RepeatModeler v. 2.0.4 (Flynn et al. 2020). We then annotated and masked repeats using RepeatMasker v. 4.1.5 (Smit et al. 2015) using the custom repeat libraries generated from RepeatModeler2 for each respective assembly. We set the search engine to “ncbi” and used the ‐xsmall option. For structural annotation of the genomes, we performed gene prediction using the web server interface https://www.plabipd.de/helixer_main.html of Helixer version v0.3.4 (Stiehler et al. 2020; Holst et al. 2023) with the lineage‐specific mode set to “invertebrate.” We used agat_sp_statistics.pl (https://agat.readthedocs.io/en/latest/why_agat.html) to calculate exhaustive statistics of the genome annotations.

We then performed a functional annotation by first conducting a BLASTP search of the predicted proteins against the ncbi‐blast protein database (Altschul et al. 1990) with an e‐value cutoff of 10^−4^ and –max_target_seqs set to 10. Then, we assigned functional annotations and Gene Ontology (GO) terms to the predicted proteins using the command line version of Blast2GO v.1.4.4 (Conesa and Götz 2008).

### 2.4 Identification, Annotation, and Comparison of the Heavy‐Chain Fibroin Silk Gene

To obtain the major silk gene of caddisflies, the *h‐fibroin* gene, we first identified the *h‐fibroin* genes in the primary and alternate assemblies of *L. insolitus* and *P. brunnea* using tBLASTn in Geneious Prime 2023.1.1 (https://www.geneious.com/). The protein sequences of the h‐fibroin N‐ and C‐termini of *Hesperophylax magnus* (Ashton et al. 2013; Heckenhauer et al. 2023; Hotaling et al. 2023) were used as query sequences for the blast search since the sequences of the N‐ and C‐termini are generally conserved in caddisflies (Heckenhauer et al. 2023). Both BLAST searches (N‐ and C‐termini queries) had hits to the same contig in the genome assemblies. We subsequently extracted the sequence between the blast hits along with 1000 bp of flanking regions from each end using the sequence view “extract” in Geneious and annotated this region in both species with Augustus v.3.4.0 (Hoff and Stanke 2019) following Heckenhauer et al. (2023). To compare the h‐fibroin among different caddisfly species, in particular those with distinct usage of silk, we extracted and annotated the *h‐fibroin* from the newly published assemblies of caddisfly species *Athripsodes cinereus* using the same approach. We then used the newly identified full‐length h‐fibroin sequences together with 11 previously published caddisfly h‐fibroin sequences (from Heckenhauer et al. 2023) to compare the primary structure, for example, repeat modules, of the h‐fibroins. For this, we visualized the repeat modules using a custom Python script (https://github.com/AshlynPowell/silk-gene-visualization/tree/main). We then calculated the amino acid composition of the h‐fibroins using Expasy ProtParam (https://web.expasy.org/protparam/, Gasteiger et al. 2005) and compared it with other Trichoptera species reported in Heckenhauer et al. (2023). To further investigate the variation in h‐fibroins among the caddisfly species, we performed hierarchical clustering analysis and principal component analysis (PCA) based on their amino acid composition. For the hierarchical clustering analysis, dissimilarities in amino acid composition were computed by calculating the Euclidean distance using the “dist” function in the Stats Package in R 4.3.3 (R Core Team 2024). The resulting dissimilarity matrix was then squared using the “ward.D2” method and subjected to hierarchical clustering with the “hclust” function in the ape package in R 4.3.3 (Paradis and Schliep 2019). The result was presented using an unrooted dendrogram. For the PCA, the amino acid composition matrix was first normalized, followed by PCA and visualization using the FactoMineR and factoextra packages in R (Lê et al. 2008).

### 2.5 Phylogenetic Reconstruction Using BUSCO Genes

To assess the phylogenetic positions of *P. brunnea* and *L. insolitus*, we reconstructed phylogenetic trees based on the BUSCO genes of 14 Trichoptera species, for which high‐quality genomes were available, using two different approaches. In the first approach, we concatenated gene alignments and subsequently estimated the tree using maximum likelihood, while in the second approach, we first inferred maximum likelihood trees for each gene and then used a multispecies coalescent approach to estimate a species tree, given that this approach is more robust to artifacts arising from incomplete lineage sorting. Single‐copy orthologs of each genome were predicted using Busco v.5.4.3 (see Section 2.2). We combined single‐copy ortholog amino acid files from each species into a single FASTA for each ortholog and aligned them using MAFFT v. 7.520 with the L‐INS‐i algorithm (Rozewicki et al. 2019). We then used Aliscore and Alicut (Misof and Misof 2009) to detect and remove the regions in the alignments that were indistinguishable from random noise. For the first approach, the cleaned alignments were concatenated into a supermatrix using FASconCAT v.1.11 (Kück and Meusemann 2010). Afterwards, we inferred a phylogenetic tree with maximum likelihood from the concatenated supermatrix using IQ‐TREE v. 2.1.3 (http://www.iqtree.org/, Minh et al. 2020). For the tree estimates, we first selected the best‐fit partitioning scheme in IQ‐TREE using the option ‐m TESTMERGEONLY. Next, we chose the optimal protein models for each metapartition with ModelFinder, as implemented in IQ‐TREE (‐m MFP, Kalyaanamoorthy et al. 2017). We performed 1000 bootstrap replicates and optimized ultrafast bootstrap trees further by applying nearest neighbor interchange based on bootstrap alignments (bnni option).

We additionally used ASTRAL‐III v. 5.7.1 for species tree inference. To do this, we first generated an individual gene tree for each locus with IQ‐TREE. The best‐fit models for each gene tree were determined using ModelFinder, as implemented in IQ‐TREE (‐m MFP), and a maximum likelihood tree was estimated with 1000 bootstrap replicates to assess branch support. We then generated the species tree from the estimated gene trees using ASTRAL‐III v. 5.7.1 (Zhang et al. 2018) with default settings.

To examine whether the h‐fibroin was potentially informative concerning phylogenetic reconstruction, we used the most conserved part of the h‐fibroin, the N‐terminus, to obtain a gene tree that is expected to resemble the species tree. For this purpose, we first aligned the N‐terminus sequences of the h‐fibroin protein (excluding signal peptides) in Geneious v. 2023.2.1 using MUSCLE 5.1 (Edgar 2022). This alignment was then used to reconstruct a maximum‐likelihood tree using the IQ‐TREE web server with default settings (http://www.iqtree.org/, Minh et al. 2020). We used three Lepidopteran species as the outgroup for the phylogenetic reconstruction: *Plodia interpunctella* (GCA_022985095.1, Kawahara et al. 2022), *Vanessa cardui* (GCA_905220365.2, Lohse et al. 2021), and *Bombyx mori* (GCA_030269925.2, Lee et al. 2024, AAF76983.1, Zhou 2000).

## 3 Results

### 3.1 Sequencing Data, Genome Assembly, and Annotation

**Figure 1:**
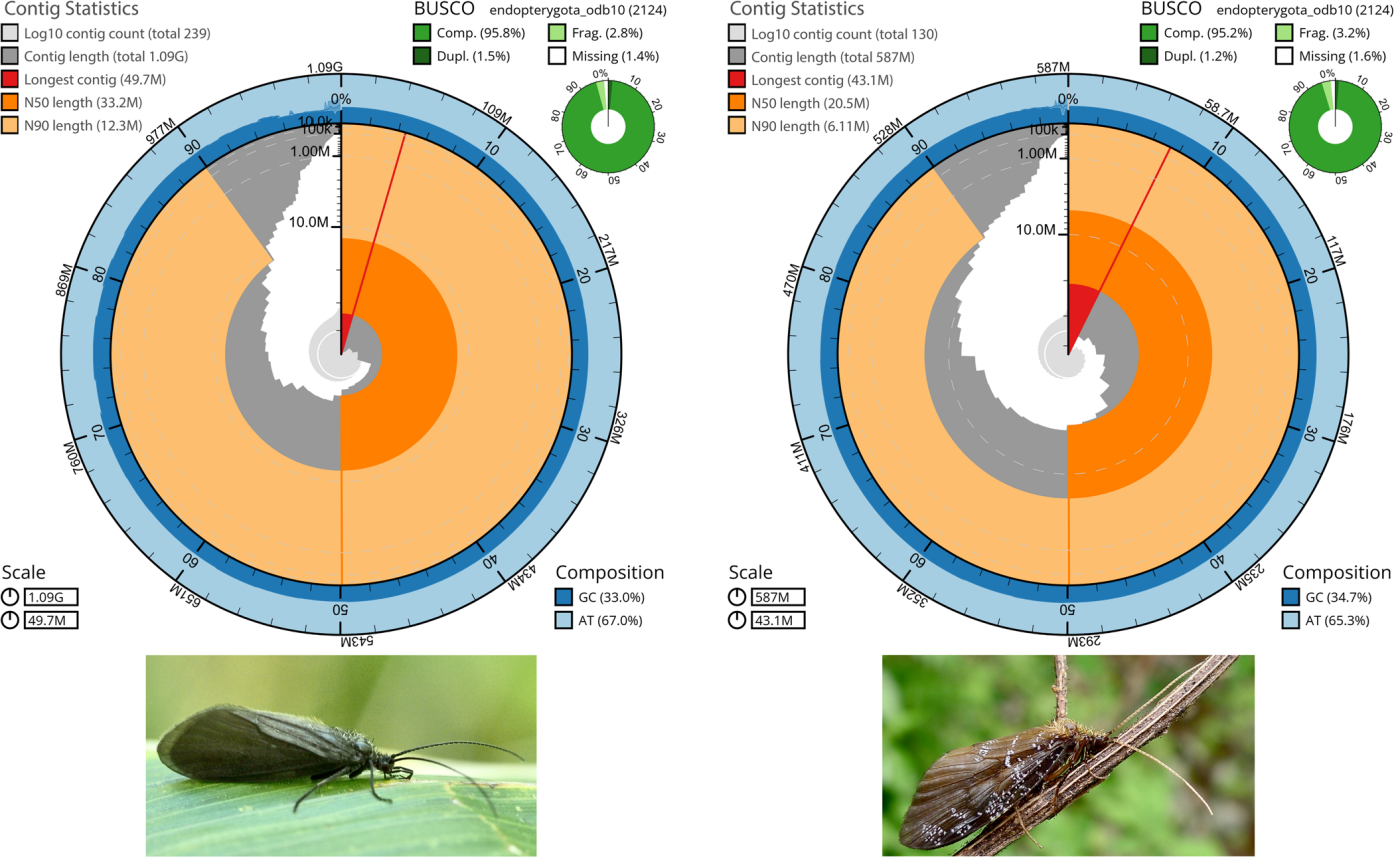
A snail plot representing genome statistics and assembly completeness for *Limnocentropus insolitus* (left) and *Phryganopsyche brunnea* (right) generated using BlobToolKit (Challis et al. 2020). The red segment represents the longest scaffold. The other scaffolds (dark gray) are arranged in size‐order moving clockwise around the plot starting from the outside of the central plot; dark orange arcs: N50 values, light orange arc: N90 values; light gray spiral: cumulative scaffold count with a white line at each order of magnitude; dark versus light blue area: mean GC versus AT content at 0.1% intervals. BUSCO scores are shown in the upper right‐hand corner. Adults of *L. insolitus* (left) and *P. brunnea* (right) were photographed by R. B. Kuranishi and H. Kurosaki, respectively.

In total, we generated 50 Gbp of HiFi data from 3,699,134 reads with an average coverage of 62× for *L. insolitus* and 12 Gbp of HiFi data from 1,306,057 reads with 25× coverage for *P. brunnea* (more details see Supporting Information S1: Table S3). The genomes presented in this study were of high contiguity, that is, a relatively large portion of the genome is assembled into long contigs. This is reflected by the high N50 values (the sequence length of the shortest contig at 50% of the total assembly length, Figure 1: dark orange arcs) relative to the length of the longest contig (Figure 1: red segment). After filtering mitochondrial contigs, the *L. insolitus* assembly contained 239 contigs with a contig N50 of 33.2 Mbp, and the *P. brunnea* assembly contained 130 contigs with a contig N50 of 20.5 Mbp (Figure 1, Table 1, Supporting Information S1: Tables S4 and S5). No contamination was detected by BlobTools for both species (Supporting Information S1: Figures S1 and S2). Re‐mapping the raw reads to the assemblies revealed that 99.91% (*L. insolitus*) and 99.95% (*P. brunnea*) of reads could be unambiguously placed. BUSCO analysis identified 95.8% (*L. insolitus*) and 95.2% (*P. brunnea*) completeness of the endopterygota OrthoDB 10 gene set in the two assemblies (Figure 1, Table 1). BlobToolKit (Challis et al. 2020) was used to visualize the quality of the genomes (Figure 1). The assembly lengths were 1,085,818,362 and 586,922,753 bp for *L. insolitus* and *P. brunnea*, respectively. The genome size estimates from GenomeScope2 were lower than the assembly length. For *L. insolitus* Genomescope2 estimated a genome size of 804 Mbp (Supporting Information S1: Figure S3). For *P. brunnea*, the genome size estimate from GenomeScope2 was 499 Mbp (Supporting Information S1: Figure S4). This discrepancy has been observed in previous studies and might be explained by the presence of repetitive elements, which could affect k‐mer estimates, resulting in smaller genome size estimates (Austin et al. 2017; Sánchez‐Herrero et al. 2019; Heckenhauer et al. 2019). In addition, a study on genome size evolution in caddisflies observed that variation among genome size estimates was higher in caddisflies with larger and more repetitive genomes, indicating issues potentially caused by repeat content (Heckenhauer et al. 2023). In fact, we observe a similar pattern in the present study. The discrepancy between the genome size estimate and assembly length is much higher in *L. insolitus* (26%) compared to *P. brunnea* (14%). The genome of *L. insolitus* is not only larger but also contains more repetitive elements.

**Table 1:** Statistics of genome assemblies of Trichoptera.

Suborder	Family	Species	Assembly length (Mbp)	Contig N50 (M)	No. of contigs	BUSCOs (*n* = 2124)	Accession number	Source
Annulipalpia	Hydropsychidae	*Arctopsyche grandis*	485.66	6.47	676	C: 97.3% [S: 93.5%, D: 3.8%], F: 1.6%, M: 1.1%	GCA_029955255.1	Frandsen et al. (2023)
Annulipalpia	Hydropsychidae	*Cheumatopsyche charites*	223.23	2.85	207	C: 96.4% [S: 95.9%, D: 0.5%], F: 1.9%, M: 1.7%	GCA_024721215.1	Ge et al. (2022)
Annulipalpia	Hydropsychidae	*Leptonema lineaticorne*	273.01	13.83	65	C: 96.1% [S: 95.3%, D: 0.8%], F: 2.3%, M: 1.6%	GCA_024500535.1	Heckenhauer et al. (2023)
Integripalpia	Hydrobiosidae	*Atopsyche davidsoni*	370.82	14.10	80	C: 96.9% [S: 96.1%, D: 0.8%], F: 1.8%, M: 1.3%	GtoCA_022113835.1	Ríos‐Touma et al. (2022)
Integripalpia	Leptoceridae	*Athripsodes cinereus*	716.26	0.95	1418	C: 95.3% [S: 94.1%, D: 1.2%], F: 2.4%, M: 2.3%	GCA_947579605.1	Wallace et al. (2023)
Integripalpia	Limnephilidae	*Glyphotaelius pellucidus*	1037.126	8.19	285	C: 90.3% [S: 89.5%, D: 0.8%], F: 6.8%, M: 2.9%	GCA_936435175.1	McSwan et al. (2023)
Integripalpia	Limnephilidae	*Hesperophylax magnus*	1215.21	11.20	980	C: 91.4% [S: 89.0%, D: 2.4%], F: 6.0%, M: 2.6%	GCA_026573805.1	Hotaling et al. (2023)
Integripalpia	Limnephilidae	*Limnephilus lunatus*	1269.657	190	139	C: 89.7% [S: 88.9%, D: 0.8%], F: 7.4%, M: 2.9%	GCA_917563855.2	Austin et al. (2023)
Integripalpia	Limnephilidae	*Limnephilus marmoratus*	1629.979	8.02	395	C: 90.4% [S: 89.3%, D: 1.1%], F: 6.7%, M: 2.9%	GCA_917880885.1	Clifford et al. (2023)
Integripalpia	Limnephilidae	*Limnephilus rhombicus*	1578.813	10.80	272	C: 89.8% [S: 88.6%, D: 1.2%], F: 7.1%, M: 3.1%	GCA_929108145.2	Broad et al. (2023)
Integripalpia	Limnocentropodidae	*Limnocentropus insolitus*	1085.82	33.23	239	C: 95.8% [S: 94.3%, D: 1.5%], F: 2.8%, M: 1.4%	JAUOEZ000000000	this study
Integripalpia	Phryganeidae	*Eubasilissa regina*	917.62	32.43	123	C: 95.5% [S: 94.8%, D: 0.7%], F: 3.0%, M: 1.5%	GCA_022840565.1	Kawahara et al. (2022)
Integripalpia	Phryganopsychidae	*Phryganopsyche brunnea*	586.92	20.54	132	C: 95.2% [S: 94.0%, D: 1.2%], F: 3.2%, M: 1.6%	JAURRE000000000	this study
Integripalpia	Rhyacophilidae	*Himalopsyche tibetana*	691.32	28.90	282	C: 96.5% [S: 95.6%, D: 0.9%], F: 2.4%, M: 1.1%	GCA_030503985.1	Heckenhauer et al. (2023)

Abbreviations: C, complete; D, duplicated; F, fragmented; M, missing; S, single.

A total of 61.96% of the *L. insolitus* genome assembly was masked as repetitive elements (tandem repeats, e.g., satellite DNA, simple repeats; and interspersed repeats, e.g., transposable elements). The majority of repeats (59.3%) were interspersed repeats of which 18.05% remained unclassified (for details, see Supporting Information S1: Table S6). In contrast, the genome assembly of *P. brunnea* contained 39.82% repeats, of which 39.06% were classified as interspersed repeats while 22.6% remained unclassified (for details, see Supporting Information S1: Table S6). The high proportion of unclassified repeats are typical for non‐model insects because of their underrepresentation in repetitive element databases (Sproul et al. 2023).

Structural annotation using Helixer identified 16,306 genes in the *L. insolitus* assembly, with an ortholog completeness of 87.6% (single copy: 86.4%, duplicated: 1.2%), as determined by BUSCO analysis. For the *P. brunnea* assembly, 17,624 genes were annotated, with a BUSCO‐determined ortholog completeness of 92.2% (single copy: 90.9%, duplicated: 1.3%, see Supporting Information S1: Table S8 for further details). Exhaustive statistics of the genome annotations are presented in Supporting Information S1: Table S7.

Functional annotation using Blast2GO revealed that, of the 16,306 sequences in the *L. insolitus* genome assembly, 9.74% were analyzed with BLAST but failed to yield significant hits. A further 15.72% returned significant alignments but could not be linked to any GO terms. Of the remaining sequences, 31.52% were mapped to GO terms but did not receive functional annotations, while 43.02% were successfully mapped and functionally annotated. For *P. brunnea*, among a total of 17,621 sequences, 15.02% yielded significant sequence alignments but could not be associated with any GO entries, 32.01% mapped to GO terms but lacked specific functional annotation assignments, and 37.2% were both mapped and functionally annotated. The remaining 15.77% were analyzed using BLAST but did not produce significant hits. The primary biological processes identified in the two genomes were cellular processes. In the molecular function category, binding and represented the largest subcategory. For the cellular component category, most genes were associated with the cellular anatomical entity (the full Blast2Go reports can be found at figshare: https://doi.org/10.6084/m9.figshare.28645466.v1).

### 3.2 Primary Structure of h‐fibroins and the Phylogenetic Position of *P. brunnea* and *L. insolitus*

The *h‐fibroin* gene and protein sequences were identified in the two new genome assemblies, as well as in the previously published *A. cinereus* assembly. As previously reported in Trichoptera (Heckenhauer et al. 2023; Frandsen et al. 2023), the *h‐fibroins* consisted of a short and a long exon and a single intron (Table 2). Further, they exhibited conserved N‐ and C‐termini and an internal region completely consisting of repeat modules. Each repeating structural module contained a characteristic, serine‐rich region (SX)nE and a glycine‐rich (cocoon and tube‐case makers) or glycine‐proline‐rich (retreat makers) region of variable length (Supporting Information S1: Figure S5). For the S(X)nE regions, S is serine, X is an aliphatic acid or arginine, *n* indicates that the SX is repeated two to six times, and E is glutamic acid.

**Table 2:** Full‐length *h‐fibroins* of three Trichoptera species newly identified in this study.

Species	Silk usage	Allele	Gene length (bp)	CDS (bp)	Exon 1 (bp)	Intron 1 (bp)	Exon 2 (bp)	Protein size (a.a.)	Molecular weight (kD)
*Limnocentropus insolitus*	Tube‐case	Primary	21,448	21,018	42	430	20,976	7005	708.836
		Alternate	21,913	21,483	42	430	21,441	7161	724.780
*Phryganopsyche brunnea*	Tube‐case	Primary	27,765	27,648	42	117	27,606	9215	872.506
		Alternate	28,167	28,050	42	117	28,008	9349	884.019
*Athripsodes cinereus*	Tube‐case	Primary	27,215	23,172	45	4043	23,127	7723	811.217
		Alternate	27,366	23,322	45	4044	23,277	7773	816.520

*Note:* Sequences are available on figshare: https://doi.org/10.6084/m9.figshare.28645466.v1.

The PacBio Revio System used for sequencing the genomes, as well as the assembly algorithm of Hifiasm allowed us to obtain both alleles of the h‐fibroin. As reported in other species (Frandsen et al. 2023), heterozygosity of h‐fibroin alleles was detected, with allele length differences of 134 amino acids in *P. brunnea* and 156 amino acids in *L. insolitus* (Table 2). In both species, we noted the presence of insertions and deletions of the repetitive modules between the two alleles (as indicated by the gray boxes in Supporting Information S1: Figure S5). The irregularly distributed insertions and deletions of repetitive modules along the h‐fibroin demonstrate variations of repeat motif content between the two alleles in the two species.

**Figure 2:**
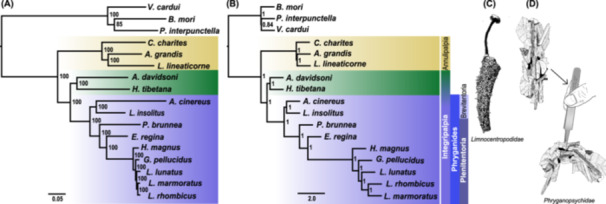
Phylogenetic trees of Trichoptera based on single BUSCO genes. (A) Phylogenetic tree inferred with IQ‐TREE. (B) Species tree inferred with ASTRAL‐III. Retreat‐making species are highlighted in yellow, cocoon‐making species in green, and tube‐case‐making species in purple rectangles. Numbers on nodes represent IQ‐TREE ultra‐fast bootstrap values and ASTRAL local posterior probabilities. The following three species of Lepidoptera are used as outgroups and were used to re‐root all the trees: *Plodia interpunctella*, *Vanessa cardui*, and *Bombyx mori*. (C) Illustration depicting the special case of Limnocentropodidae, and (D) the special case of Phryganopsychidae, illustrating that the case bends under its own weight when grasped by forceps. Illustrations were created by Ralph W. Holzenthal.

To explore the phylogenetic relationships of the Trichoptera species in this study, we estimated phylogenetic trees using the BUSCO gene sequences. The phylogenetic relationships revealed in these results were consistent with recently published phylogenetic hypotheses (Thomas et al. 2020; Frandsen et al. 2024): (1) Trichoptera is divided into two suborders: Annulipalpia (retreat makers, Figure 2A,B: yellow) and Integripalpia (consisting of cocoon makers, Figure 2A,B: green and tube‐case makers (infraorder Phryganides), Figure 2A,B: purple). As expected, *P. brunnea* and *L. insolitus* were included in the clade containing the other tube‐case‐making species (Figure 2A,B: purple). (2) Phryganides are divided into Plenitentoria and Brevitentoria. Within Plenitentoria, Phryganopsychidae, consisting of the sole genus *Phryganopsyche*, appears as sister taxon to the remaining Plenitentoria, while Limnocentropodidae (with genus *Limnocentropus*) forms a clade with the other species of Brevitentoria (*A. cinereus*, family Leptoceridae). The gene tree based on the alignment of the conserved N‐terminus of the h‐fibroin (126 amino acid residues) was congruent with the species trees in the sense that it exhibits the same main three clades (retreat, cocoon, and tube‐case makers, Supporting Information S1: Figures S6 and S7). However, most of the nodes, including the positions of *P. brunnea* and *L. insolitus* had low bootstrap support. The alignment likely contained too few informative sites to generate high branch support.

### 3.3 Amino Acid Composition of h‐fibroins

**Figure 3:**
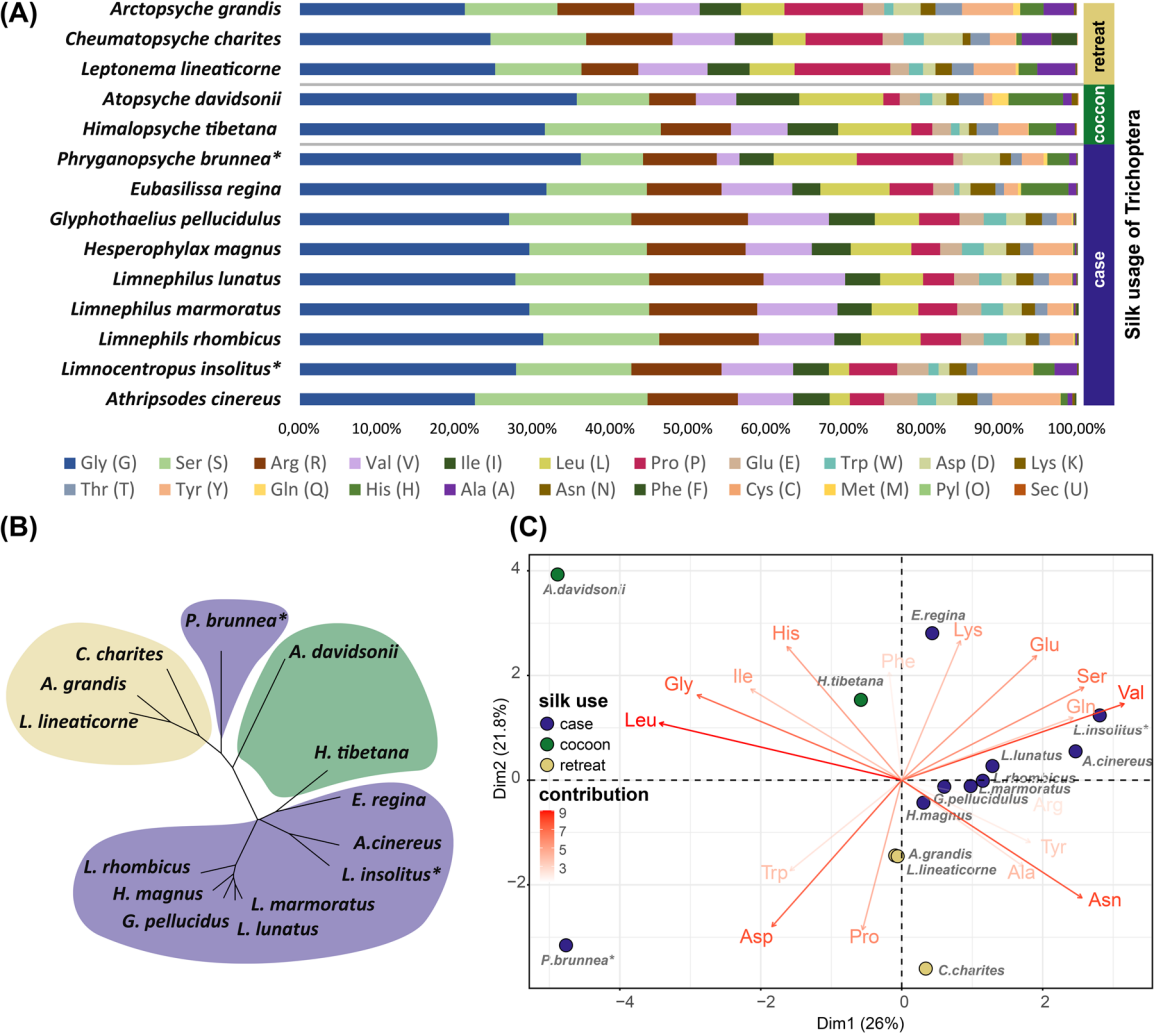
Amino acid composition of h‐fibroins of Trichoptera species with different silk usage (A), the cluster of unrooted dendrogram built with amino acid composition (B), and principal component analyses (PCA) based on amino acid composition (C). Species in panel A are ordered (from top to bottom) according to the phylogenetic relationships estimated by (Frandsen et al. 2024). *h‐fibroins generated in this study. Branches of the dendrogram are colored according to phylogenetic placement: yellow: Annulipalpia, green: cocoon‐making Integripalpia, purple: case‐making Integripalpia. The colors of the patches in panel C represent the silk use of each individual. The eigenvalues of the corresponding component are shown on the axis labels.

To study the variation of h‐fibroin across different silk‐usages, we compared the amino acid composition among case‐, cocoon‐, and retreat‐making species. In general, glycine and serine together show the highest proportion across all Trichoptera species (33.1%–46.4%, Figure 3, Supporting Information S1: Figure S8). Nonetheless, the proportion of certain amino acids differed among taxa. For instance, as observed in a previous study (Heckenhauer et al. 2023), the retreat‐making caddisfly species (9.9%–12.3%) included a higher proline proportion than tube‐case‐ (3.7%–6.2%) and cocoon‐making (2.1%–2.7%) caddisflies (Figure 3A). However, in contrast to the other tube‐case‐making caddisflies, *P. brunnea* exhibited a markedly elevated proportion of proline (12.4%), more similar to retreat‐making caddisflies. In addition, together with the two cocoon‐making species, it exhibited the highest fraction of leucine. The amino acid composition of the h‐fibroin of *L. insolitus* was generally similar to other tube‐case‐making species. However, the proportion of alanine which constitutes a large proportion of lepidopteran h‐fibroins was notably higher in the h‐fibroin of *L. insolitus* (2.9%) than that observed in other tube‐case‐making species (0.1%–1.0%) and cocoon (1.1%–2.4%), but lower than in retreat‐making species (3.8%–4.9%).

We performed a hierarchical clustering analysis based on the distance matrix of the amino acid composition of the h‐fibroin and generated a dendrogram to show the similarity and dissimilarity of the amino acid composition of the h‐fibroin across all caddisfly species (Figure 3B). The clusters shown in the dendrogram generally align with the phylogenetic relationships reconstructed from BUSCO genes (Figure 2). Specifically, all retreat‐making species were clustered together. Moreover, the tube‐case‐making species form a large cluster with the notable exception of *P. brunnea*, which was separated from tube‐case makers, suggesting that the amino acid composition of *P. brunnea* was more similar to retreat‐making species than to tube‐case makers. Within tube‐case makers, the five most closely related species (three *Limnephilus* species, *H. magnus* and *Glyphotaelius pellucidus*), as well as *L. insolitus* and *A. cinereus* (both Brevitentoria) form stable associations. To further investigate this difference and to capture the most important amino acids, we performed PCA to identify clusters of species based on their amino acid composition. The first two components (PC1 and PC2) explained 47.8% of the total variance (Figure 3C, Supporting Information S1: Figure S9). According to the principal component loadings in the plot, the amino acids leucine, valine, and glycine had high loading on PC1, while proline, aspartic acid, and lysine had high loadings on PC2. In general, the results reflect the hierarchical clustering analysis. In line with the dendrogram, *P. brunnea* was separated from tube‐case makers.

While the placement of *P. brunnea* in the cluster dendrogram and PCA analysis diverges most strongly from the phylogenetic results, there are also a few other shifts that are worth mentioning. For example, *Himalopsyche tibetana* is more strongly clustered with another tube‐case maker, *Eubasilissa regina*, than it is with the other cocoon‐making species (*Atopsyche davidsoni*).

## 4 Discussion

In this study, we generated high‐quality genome assemblies and annotations for two caddisfly species with unique case‐making behaviors. With advancements in sequencing technologies and computational tools, extensive high‐quality reference genome resources have been developed in recent years. However, to date, genomes of only 41 caddisfly species have been sequenced, representing merely ~0.3% of the total caddisfly diversity. Our high‐quality genome assemblies of two caddisfly species enhance these resources, laying a robust foundation for future studies on caddisfly evolution. Moreover, retrieving the complete *h‐fibroin* genes from these assemblies enables us to compare gene evolution across species with diverse silk usage, thereby offering new insights into the genetic basis of the evolutionary innovation of retreat‐ and case‐making.

Phylogenetic reconstruction using BUSCO genes from 14 high‐quality genomes of caddisfly species spanning all three clades revealed that, consistent with their case‐making behavior, *P. brunnea* and *L. insolitus* cluster with other tube‐case makers, in line with recent comprehensive phylogenomic studies of caddisflies (Frandsen et al. 2024). Although phylogenetically classified as a tube‐case maker, the silk of *P. brunnea* exhibits distinct characteristics. Previous observations have noted that the larval cases of *P. brunnea* are unusually long and floppy, bending under their own weight when removed from water, and resemble annulipalpian retreats in structure (Wiggins 2004). This divergence from tube‐case‐making species is also evident in the primary structure of the major silk gene, *h‐fibroin*. While the similarities between h‐fibroins estimated from the hierarchical cluster analysis of h‐fibroin amino acid composition largely align with phylogenetic relationships—such as *L. insolitus* clustering with other tube‐case makers and species from the same clades grouping together—it also highlights a notable exception: *P. brunnea* is separated from the other tube‐case‐making species. While amino acid composition generally mirrors suborder phylogeny, *P. brunnea* stands out among tube‐case makers, largely due to a proline content (12.4%) that is significantly higher than that of other tube‐case‐making caddisflies (3.7%–6.2%) and comparable to retreat makers (9.9%–12.3%). Proline has been hypothesized to enhance the extensibility of silk fibers by increasing secondary structure disorder in the amorphous regions of silk, as observed in spiders where this trait facilitates the capture of flying prey without breaking the web (Liu et al. 2008; Marhabaie et al. 2014; Rauscher et al. 2006; Savage and Gosline 2008a, 2008b). The flexible retreats of the retreat‐making species presumably require more extensible silk compared to the rigid tube‐cases. The shared silk composition between *P. brunnea* and retreat makers, particularly the elevated proline content, suggests functional convergence potentially linked to the mechanical demands of their silk structures.

The *P. brunnea* h‐fibroin was not the only sequence that was divergent from the species tree. For example, the h‐fibroin sequences of cocoon‐making species *H. tibetana* and *A. davidsoni* also did not cluster together. Rather, *H. tibetana* clustered with tube‐case maker, *E. regina*. Better understanding this relationship would be an interesting avenue for future research.

In conclusion, the high‐quality genome assemblies generated in this study provide valuable insights into the relationship between case‐making behavior and silk gene composition in caddisflies, particularly in the unusual case‐making species *P. brunnea*. Our findings reveal that, while the BUSCO gene phylogenetic analysis shows that *P. brunnea* is most closely related to other tube‐case makers, the amino acid composition of the primary silk gene, especially the elevated proline content, more closely resembles that of retreat makers. This highlights the complexity of silk adaptation across different caddisfly species. However, to fully understand the functional implications of these genetic differences, studies on mechanical properties of silk fibers are urgently needed. Such investigations will be crucial for linking genetic variation to the mechanical properties of silk and its ecological adaptations.

## Author Contributions

J.H., P.B.F., and S.U.P. designed the study and acquired funding. R.B.K. collected and preserved the specimens. P.B.F. performed the genome sequencing and assembly. X.D., J.H., and P.B.F. analyzed and curated the data. X.D. visualized the data. X.D. and J.H. wrote the original draft of the manuscript. All authors contributed to editing and revising the manuscript.

## Conflicts of Interest

The authors declare no conflicts of interest.

## Supporting information

supplementary_material_revision.

Limnocentropus_insolitus_movie.

## Data Availability

The raw sequence reads are available at the Sequence Read Archive (SRA) with accession numbers SRR25447064 and SRR25365886. The final genome assembly is available at the SRA with accession numbers JAURRE000000000 (*P. brunnea*) and JAUOEZ000000000 (*L. insolitus*). Other supporting information is presented in the supporting material. Alternate assemblies, repeat‐masked assemblies, mitogenome assemblies, structural (Helixer proteins fasta and gtf file) and functional annotations (xml files with blasted proteins and Blast2GO reports), as well as the *h‐fibroin* sequences of *P. brunnea* and *L. insolitus*, along with the protein sequence of h‐fibroin from other species used in this study, have been deposited at figshare under the following link: Heckenhauer et al. (2025). Additional Files for “De novo whole genome assemblies of unusual case‐making caddisflies (Trichoptera) highlight genomic convergence in the composition of the major silk gene (h‐fibroin).” Figshare data set: https://doi.org/10.6084/m9.figshare.28645466.v1.
