# Supplementary material for: De Novo Whole Genome Assemblies of Unusual Case‐Making Caddisflies (Trichoptera) Highlight Genomic Convergence in the Composition of the Major Silk Gene (h‐fibroin)
Source: J Exp Zool B Mol Dev Evol. 2025 May 19;344(7):442–53. doi: 10.1002/jez.b.23301 (PMC12576367; doi:10.1002/jez.b.23301)
Supplement: Supplementary file 1 — supplementary_material_revision. [file JEZ-344-442-s001.docx]

Supplementary Material

**Supplementary Note 1**

Because of the live stage of *L.insolutis* specimen (larva) sex determination was not possible based on the genitals. Since Trichoptera follows the ZO female sex determination system, contigs with half the sequencing depth should be assigned to female sex chromosomes. Interestingly, the blobtools coverage plots (supp.fig. S1 & S2) both show a major and some smaller contigs about half-coverage relative to the bulk of the genome. This pattern is typical for the haploid Z chromosome from female samples. To confirm that both specimens are female, we downloaded the closest related available chromosome length reference genomes (*Athripsodes cinereus,* GCA_947579605.1 for *L. insolitus* and *Limnephilus lunatus*, GCA_917563855.2 for *P. brunnea*) and used ragtag v2.1.0 *scaffold* with default settings to align our assembled contigs to chromosomes. We then mapped back our raw reads to the genome assemblies and calculated coverage with qualimap v2.3 *bamqc -bam*. We compared the coverage stats of the contigs that aligned to the Z chromosome to those of the top ten longest contigs that aligned to the autosomal contigs of the reference genomes (see Tables S1 & S2). In the end, we observed approximately half of the sequencing coverage for the contigs that aligned to the Z chromosome, consistent with the female ZO karyotype. This is strong evidence that the samples were female.

**Supplementary Table S1:** Sequencing coverage stats of the contigs of the *P. brunnea* assembly that aligned to the Z chromosome of the *Limnephilus lunatus* genome, as well as top ten longest contigs of the *P. brunnea* assembly that aligned to the autosomes of the *Limnephilus lunatus* genome.

| contig | length | mapped | mean cov | std cov | aligned to |
| --- | --- | --- | --- | --- | --- |
| ptg000012l | 11665986 | 133985018 | 11.4851002 | 3.67367434 | Z chromosome |
| ptg000015l | 438593 | 5325802 | 12.1429252 | 7.60623722 | Z chromosome |
| ptg000021l | 8497733 | 99401263 | 11.697386 | 3.61062182 | Z chromosome |
| ptg000027l | 3881938 | 40789112 | 10.5074094 | 3.30967598 | Z chromosome |
| ptg000032l | 3429073 | 34173347 | 9.9657683 | 3.37128496 | Z chromosome |
| ptg000036l | 3199464 | 36181811 | 11.3087102 | 3.63412947 | Z chromosome |
| ptg000051l | 2328564 | 23156542 | 9.94455896 | 3.4819827 | Z chromosome |
| ptg000083l | 207629 | 2190355 | 10.5493693 | 3.1038561 | Z chromosome |
|  |  |  |  |  |  |
| ptg000002l | 43145923 | 961088884 | 22.2753117 | 6.02598843 | autosome |
| ptg000029l | 35604711 | 789014256 | 22.1603893 | 6.26557942 | autosome |
| ptg000016l | 33734492 | 767958875 | 22.7647974 | 6.24911573 | autosome |
| ptg000013l | 33140845 | 717255952 | 21.6426573 | 6.00438295 | autosome |
| ptg000006l | 30377984 | 695067408 | 22.8806299 | 6.09180131 | autosome |
| ptg000007l | 29586023 | 660235065 | 22.3157761 | 6.27710461 | autosome |
| ptg000001l | 25809718 | 583983152 | 22.6264832 | 5.95597111 | autosome |
| ptg000014l | 22050876 | 505060191 | 22.9043141 | 6.15430674 | autosome |
| ptg000005l | 21029621 | 488001693 | 23.205444 | 5.84225005 | autosome |
| ptg000028l | 20543509 | 443055294 | 21.5666805 | 5.95208692 | autosome |

**Supplementary Table S2:** Sequencing coverage stats of the contigs of the *L.insolitus* assembly that aligned to the Z chromosome of the *Limnephilus lunatus* genome, as well as top ten longest contigs of the *L.insolitus* assembly that aligned to the autosomes of the *Limnephilus lunatus* genome.

| contig | length | mapped | mean cov | std cov | aligned to |
| --- | --- | --- | --- | --- | --- |
| lcl\|ptg000003l | 48889023 | 1206246687 | 24.67316 | 7.62032651 | Z chromosome |
|  |  |  |  |  |  |
| lcl\|ptg000005l | 49656299 | 2288797716 | 46.0927971 | 12.9886044 | autosome |
| lcl\|ptg000004l | 47789013 | 2304780995 | 48.2282611 | 11.7490676 | autosome |
| lcl\|ptg000023l | 45047504 | 2166705372 | 48.0982336 | 12.1612132 | autosome |
| lcl\|ptg000009l | 44634295 | 2212876504 | 49.5779423 | 12.2912846 | autosome |
| lcl\|ptg000019l | 42536382 | 2059215104 | 48.4106783 | 11.3352807 | autosome |
| lcl\|ptg000002l | 42452035 | 2009650285 | 47.3393157 | 11.6010361 | autosome |
| lcl\|ptg000036l | 36844112 | 1749072310 | 47.4722341 | 12.0882926 | autosome |
| lcl\|ptg000008l | 36604506 | 1795200953 | 49.0431684 | 11.5672196 | autosome |
| lcl\|ptg000013l | 36518729 | 1741545754 | 47.6891119 | 11.7363892 | autosome |
| lcl\|ptg000016l | 36457263 | 1735768701 | 47.6110536 | 12.1737169 | autosome |


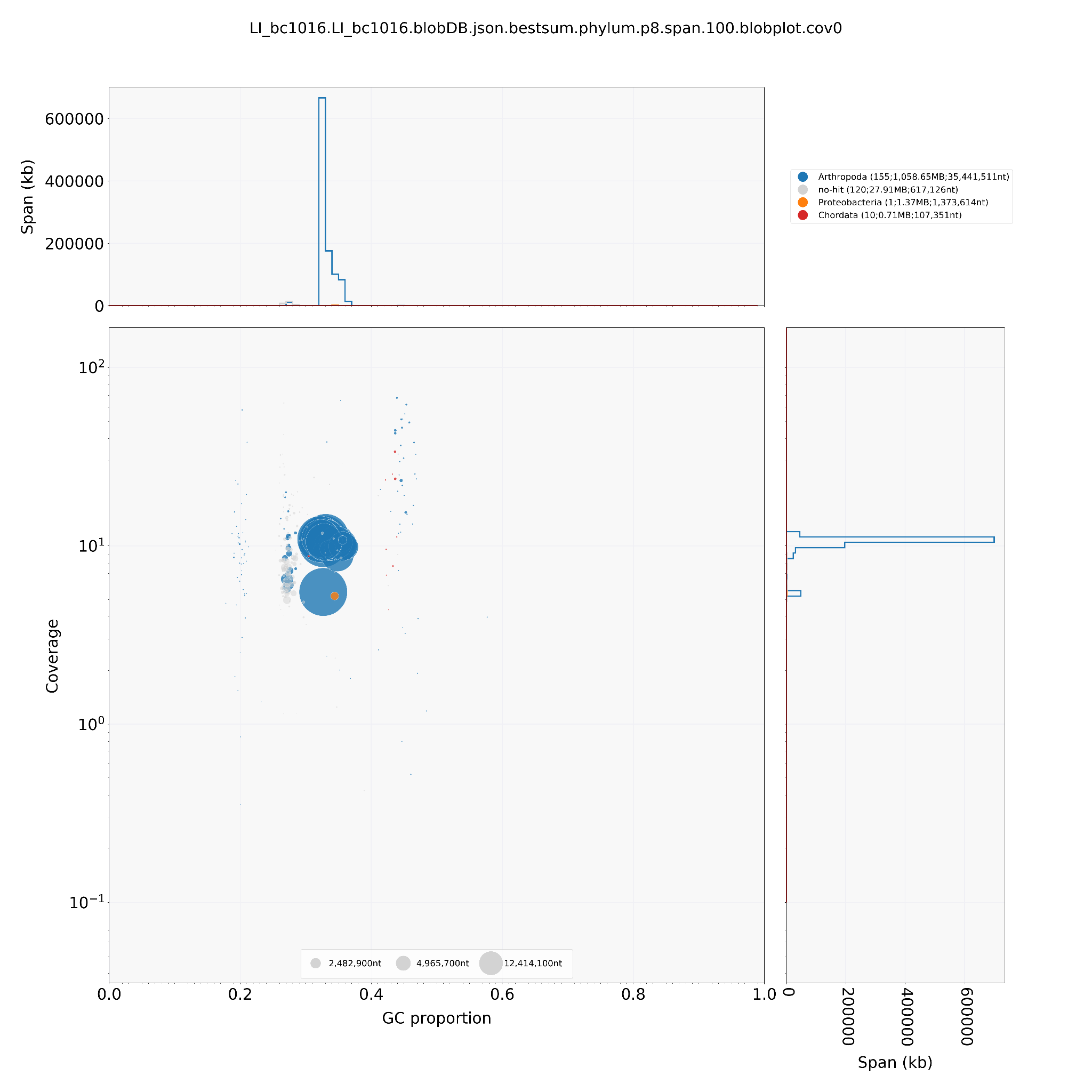


**Figure S1** Taxon-annotated GC-coverage (TAGC) plots of *L. insolitus* genome assembly. Circles in the scatter plot represent contigs (with diameters proportional to sequence length) and are coloured by taxonomic affiliation (blue: Arthropoda, orange: Proteobacteria, red: Chordata, grey: no hits). The position of the circles on the y-axis is based on the base coverage of the sequence in the coverage library. The position of the circles on the x-axis is based on the GC content. The upper and right hand panels coverage and GC histograms for each taxonomic group, which are weighted by the total span (cumulative length) of sequences occupying each bin. The description next to the taxonomic affiliation of sequences (on the right in the top) lists count, total span and N50 by taxonomic group.


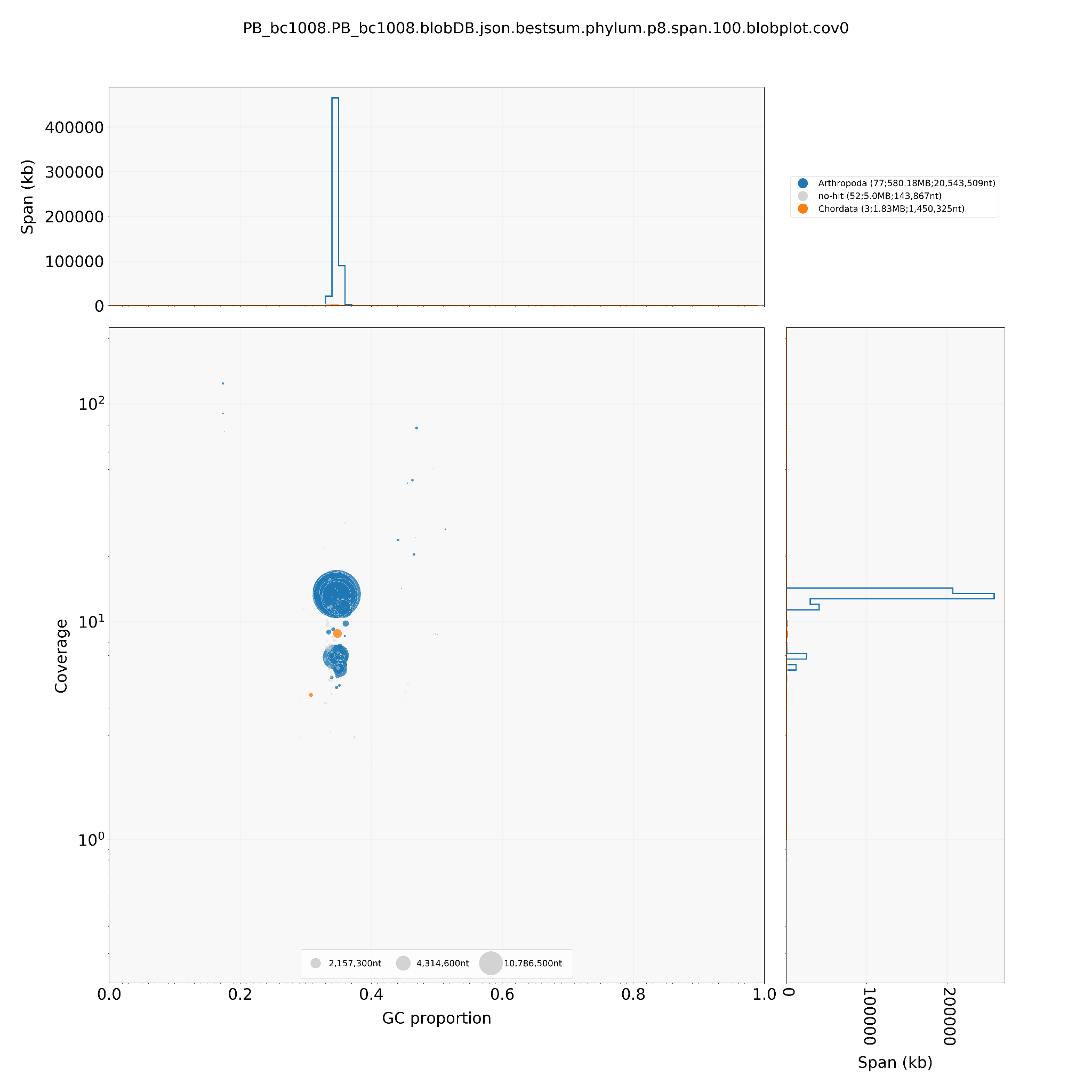


**Figure S2** Taxon-annotated GC-coverage (TAGC) plots of *P. brunnea* genome assembly. Circles in the scatter plot represent contigs (with diameters proportional to sequence length) and are coloured by taxonomic affiliation (blue: Arthropoda, orange: Chordata, grey: no hits). The position of the circles on the y-axis is based on the base coverage of the sequence in the coverage library. The position of the circles on the x-axis is based on the GC content. The upper and right hand panels coverage and GC histograms for each taxonomic group, which are weighted by the total span (cumulative length) of sequences occupying each bin. The description next to the taxonomic affiliation of sequences (on the right in the top) lists count, total span and N50 by taxonomic group.


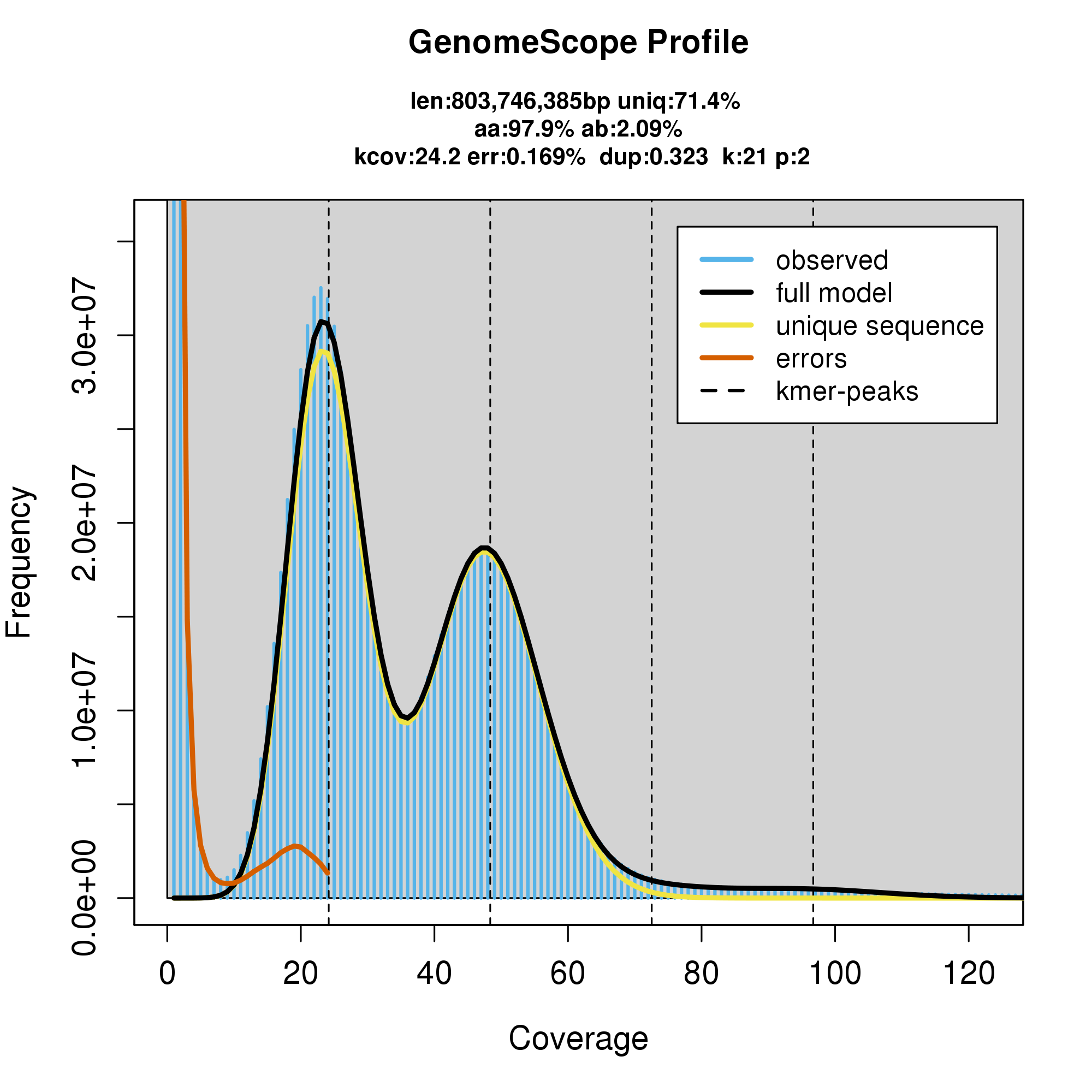

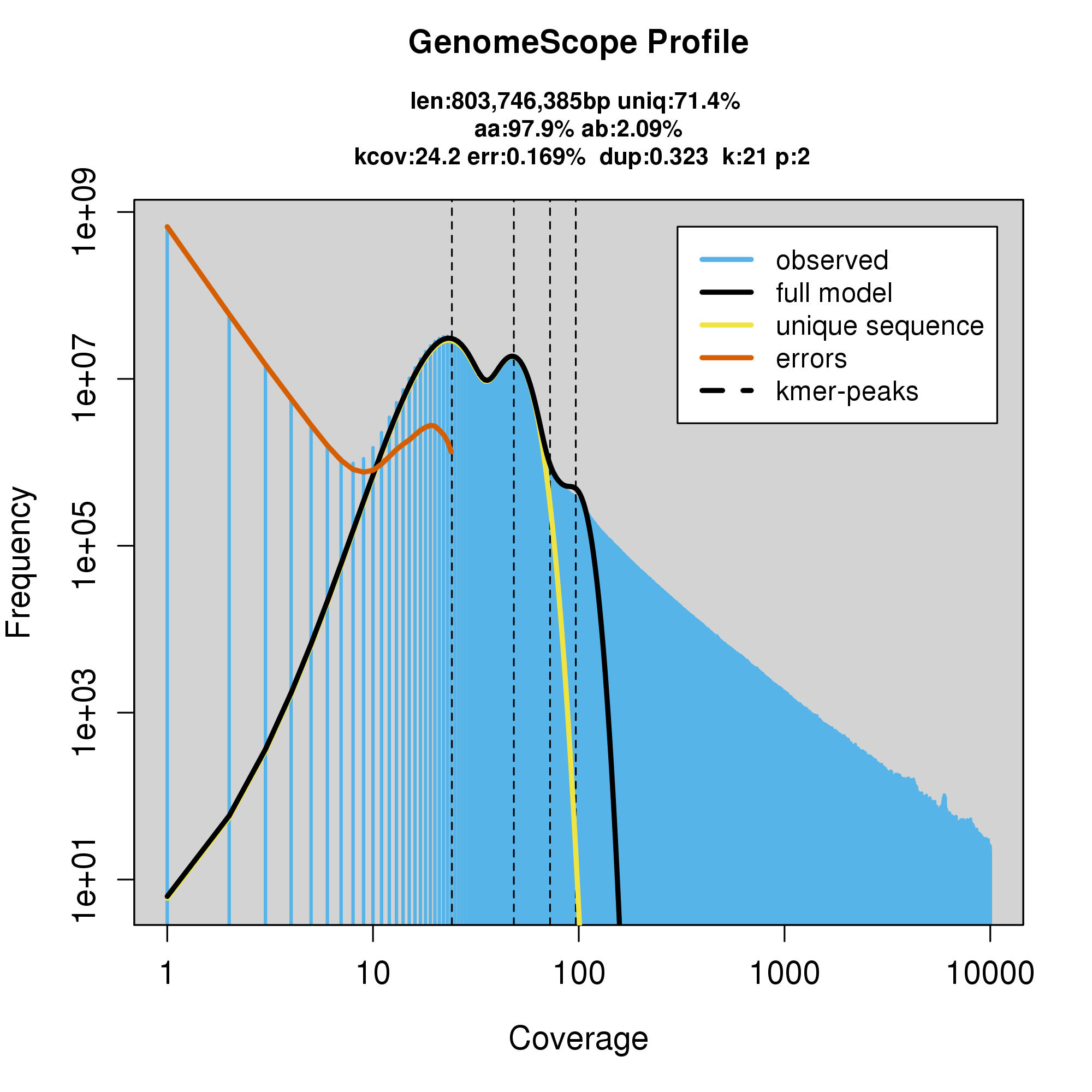


**Figure S3** Genomescope2 profiles for *Limnocentropus insolitus*. Left linear plot Right log plot; len: inferred total genome length, uniq: percent of the genome that is unique (not repetitive), kcov: mean k-mer coverage for heterozygous bases, err: error rate of the reads, dup: average rate of read duplications.


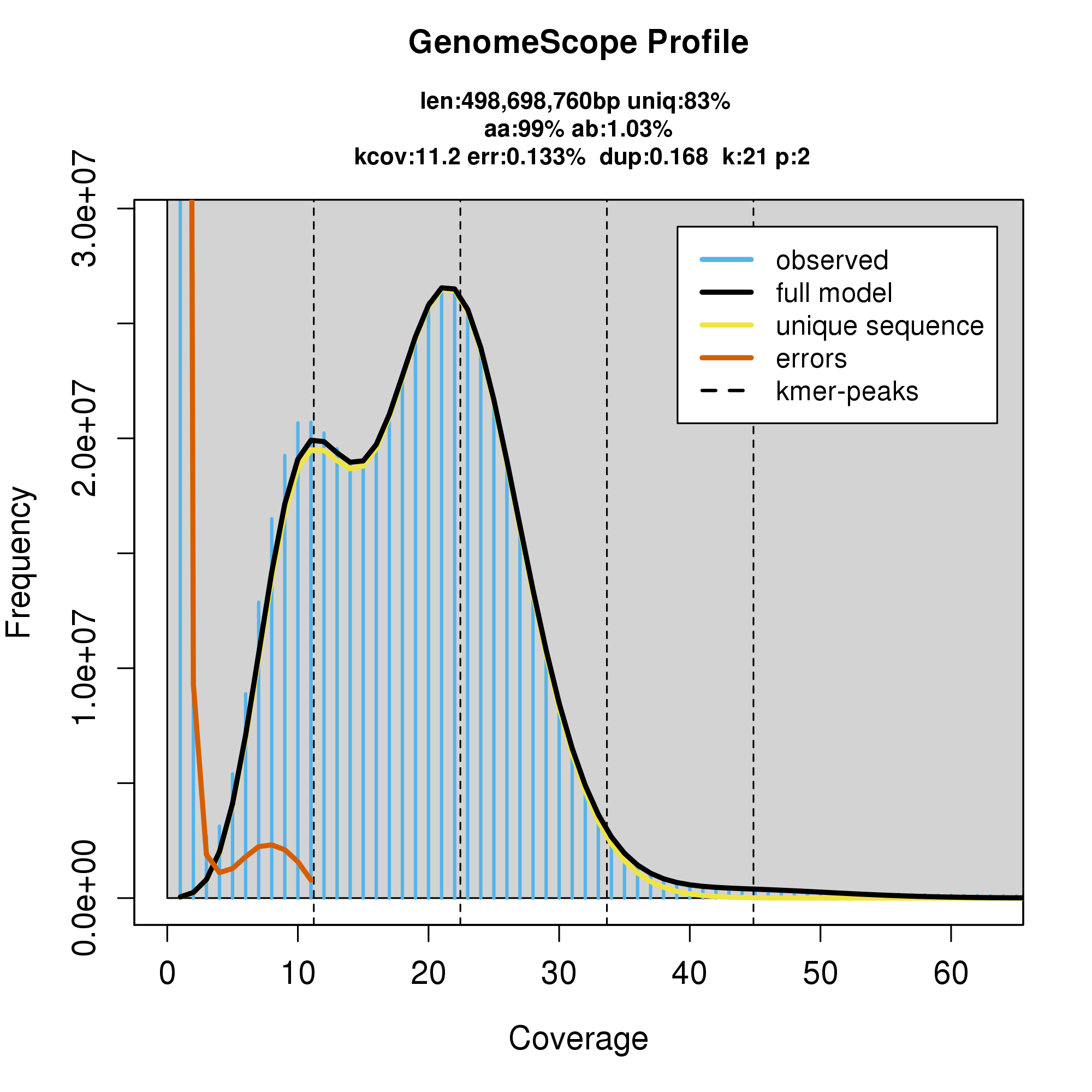

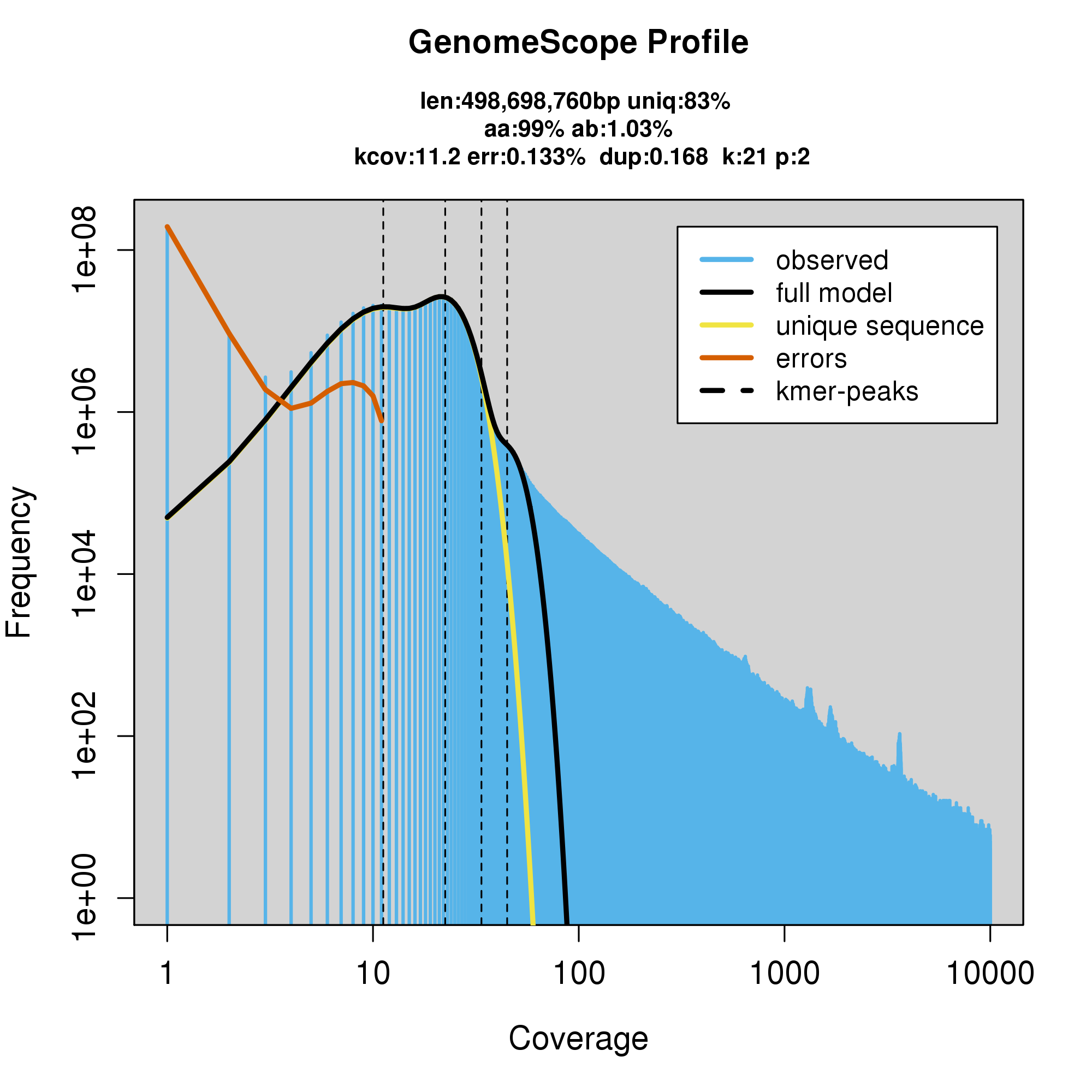


**Figure S4** Genomescope2 profiles for *Phryganopsyche brunnea*. Left linear plot Right log plot; len: inferred total genome length, uniq: percent of the genome that is unique (not repetitive), kcov: mean k-mer coverage for heterozygous bases, err: error rate of the reads, dup: average rate of read duplications.


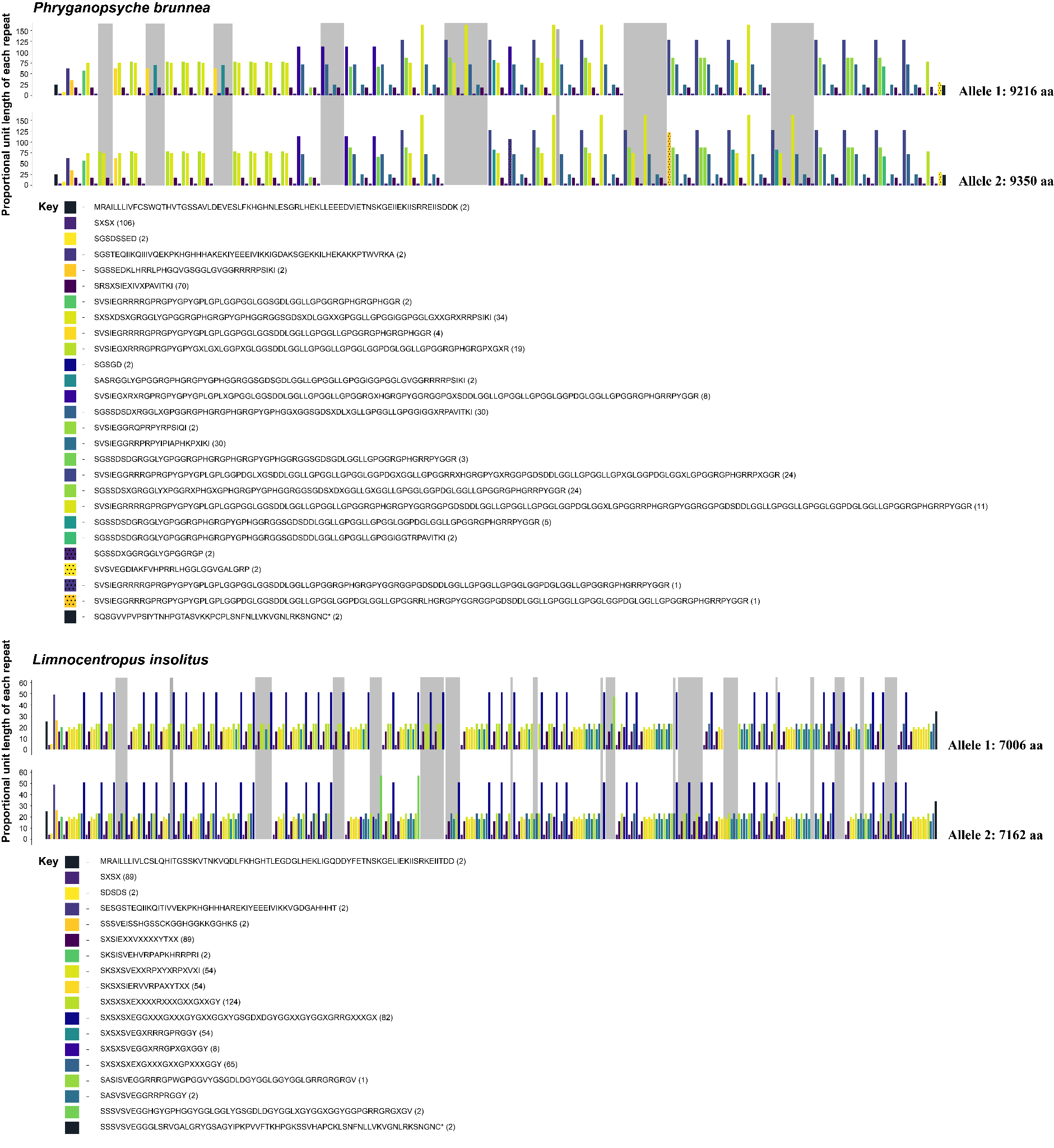
**Figure S5** Repeat module of the allelic h-fibroin silk gene of the two caddisfly species. Bars of the same color represent the same repetitive motif within an individual, but not among individuals. The height of the bars indicates the proportional unit length of each repeat. The grey boxes show insertions and deletions between alleles. The total length of protein sequences is noted on the right side of the figure.


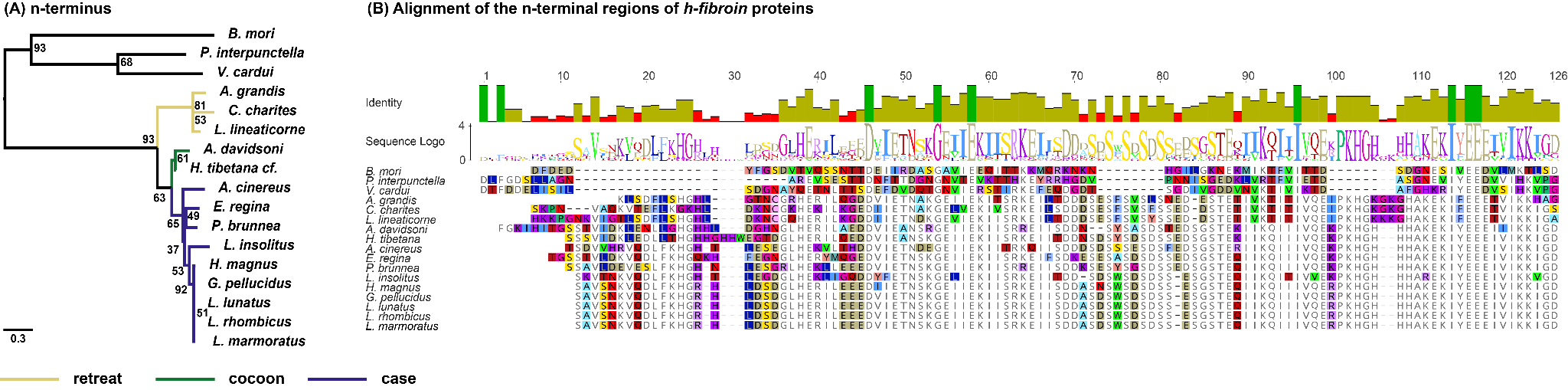


**Figure S6** Maximum-likelihood tree of Trichoptera species used in this study based on the N-terminus alignment of the h-fibroin protein (excluding signal peptides) (A) and alignment of the conserved N-terminal regions of h-fibroin proteins (B). The ML tree was estimated in IQ-TREE. Numbers on nodes represent ML IQ-TREE 1000 bootstrap values. The following three species of Lepidoptera are used as outgroups and were used to re-root all the trees: .*Plodia interpunctella*, *Vanessa cardui*, and *Bombyx mori*. The amino acid sequence logos show conservation patterns in the protein alignment.


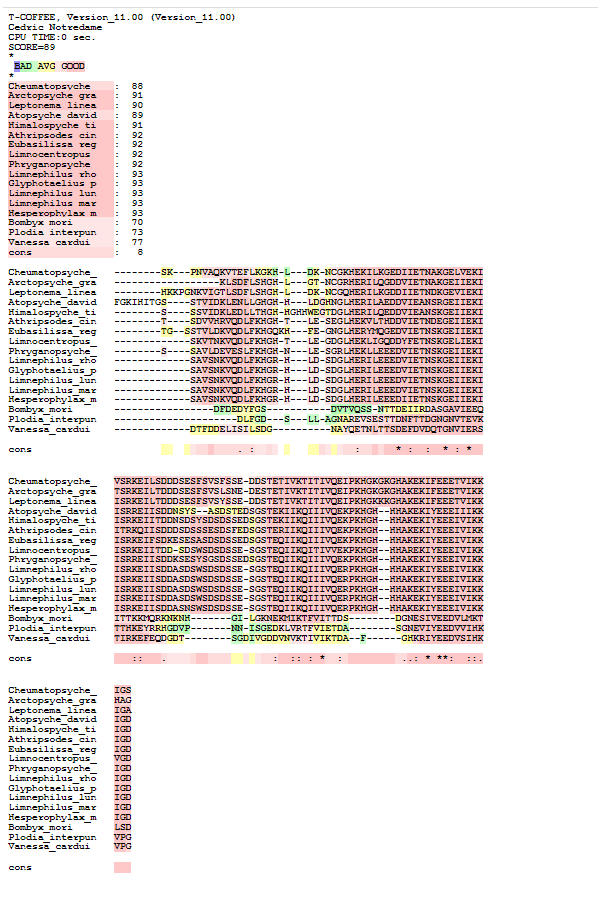


**Figure S7** T-coffe alignment of N-Terminus.


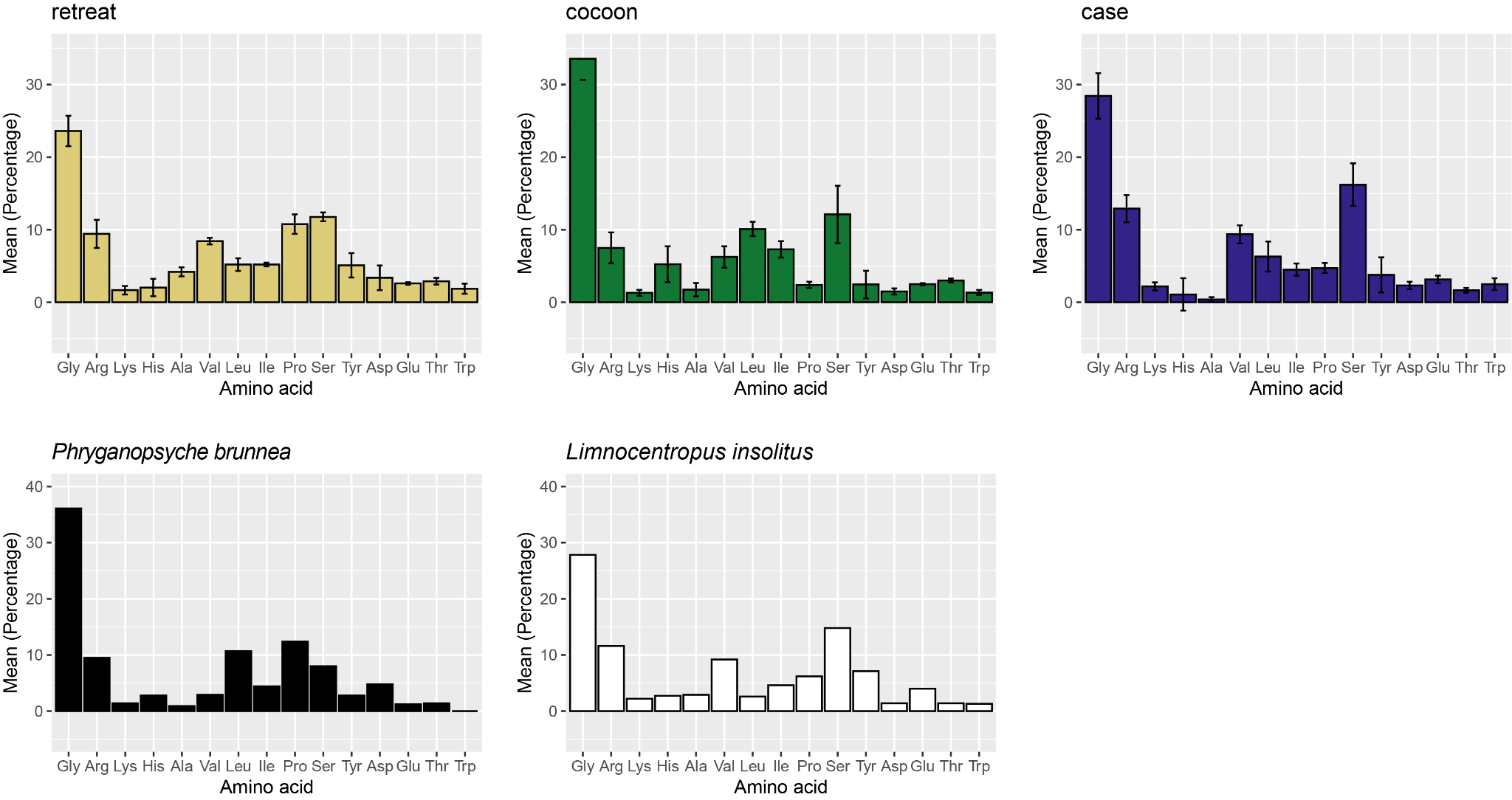


**Figure S8** The mean amino acid composition of the *h-fibroins* silk gene of the three Trichoptera groups and the two focal species in this study.


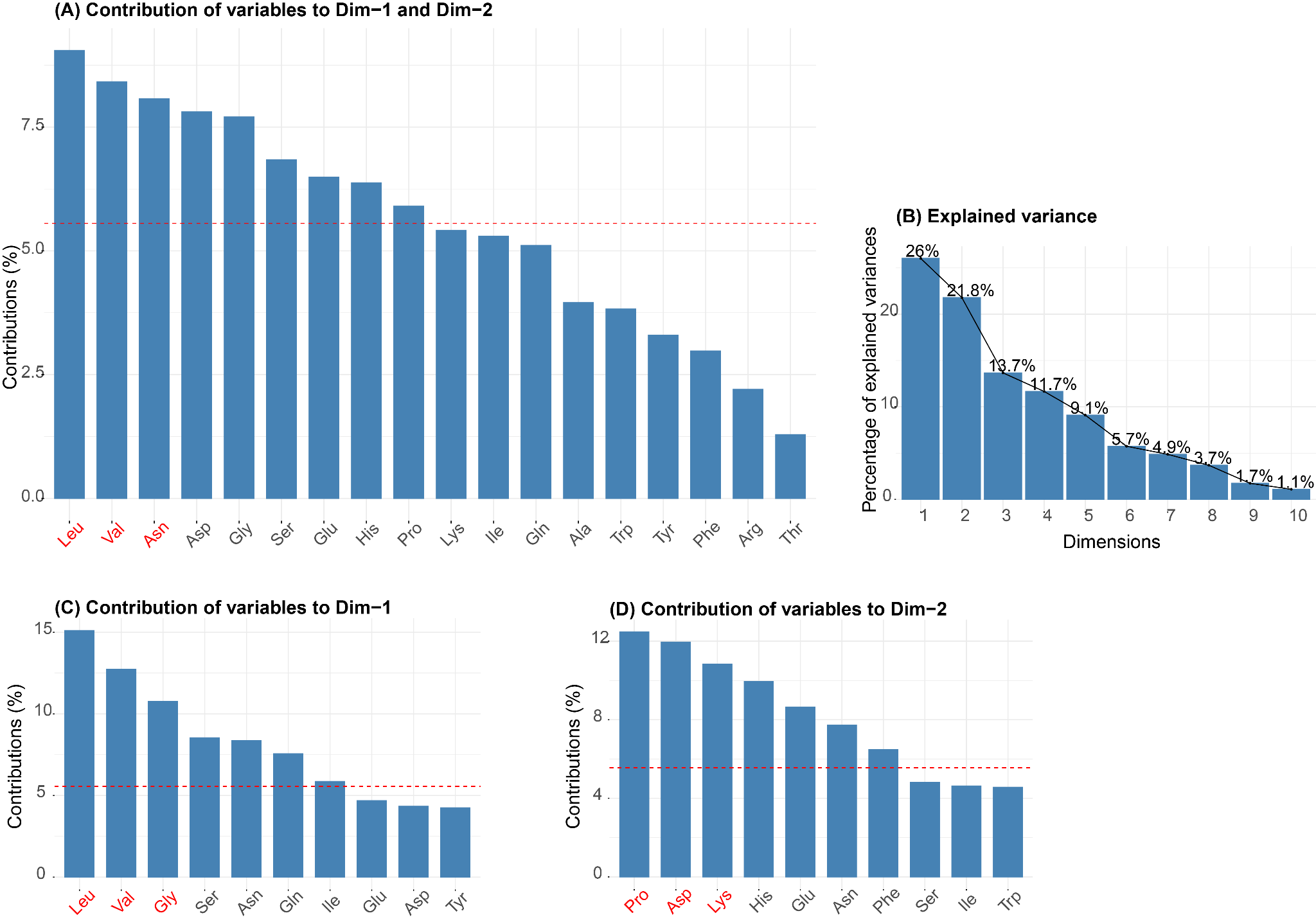


**Figure S9** Statistics of the PCA analysis. (A) Contribution of amino acid composition to dimensions 1 and 2, (B) the explained variance, (C) contribution of amino acid composition to dimension 1, (D) contribution of amino acid composition to dimension 2. The red dashed lines indicated the expected average contribution. For a given component, the top three contributing variables were highlighted in red color.

**Supplementary Table S3** Basic statistics of raw genome data of *L. insolitus* and *P. brunnea*

| Assembly | *L. insolitus* | *P. brunnea* |
| --- | --- | --- |
| Total Sequence | 3,699,134 | 1,306,057 |
| N50 | 13,627 | 10,744 |
| Average Length | 13,547 | 9,557 |
| Median length | 12,412 | 8,67 |
| Total Length | 50,112,654,364 | 12,483,219,597 |
| Shortest Length | 61 | 61 |
| Longest Length | 60,147 | 48,814 |
| Coverage | 62× | 25× |

* Statistics were estimated by fast_stats.py (https://github.com/sandyjmacdonald/fast_stats)

**Supplementary Table S4** Statistics of genome assemblies of *L. insolitus* and *P. brunnea* before filtering mitochondrial and contaminated contigs

| Assembly | *L. insolitus* | *P. brunnea* |
| --- | --- | --- |
| Contigs (>=0bp) | 286 | 132 |
| Contigs (>=1000bp) | 286 | 132 |
| Contigs (>=5000bp) | 286 | 132 |
| Contigs (>=10000bp) | 285 | 131 |
| Contigs (>=25000bp) | 266 | 118 |
| Contigs (>=50000bp) | 159 | 105 |
| Total length (>=0bp) | 1088643004 | 587009648 |
| Total length (>=1000bp) | 1088643004 | 587009648 |
| Total length (>=5000bp) | 1088643004 | 587009648 |
| Total length (>=10000bp) | 1088635537 | 586999772 |
| Total length (>=25000bp) | 1088240803 | 586780407 |
| Total length (>=50000bp) | 1084509907 | 586307413 |
| contigs | 286 | 132 |
| Largest contig | 49656299 | 43145923 |
| Total length | 1088643004 | 587009648 |
| GC (%) | 33.00 | 34.69 |
| N50 | 33230923 | 20543509 |
| L50 | 14 | 10 |
| N's per 100 kbp | 0.00 | 0.00 |

* Assembly stats were calculated using quast (Trizna, 2020). All statistics are based on contigs of size >= 500 bp, unless otherwise noted (e.g., "contigs (>= 0 bp)" and "Total length (>= 0 bp)" include all contigs).

**Supplementary Table S5** Statistics of genome assemblies of *L. insolitus* and *P. brunnea* after filtering mitochondrial and contaminated contigs

| Assembly | *L. insolitus* | *P. brunnea* |
| --- | --- | --- |
| Contigs (>=0bp) | 239 | 130 |
| Contigs (>=1000bp) | 239 | 130 |
| Contigs (>=5000bp) | 239 | 130 |
| Contigs (>=10000bp) | 239 | 129 |
| Contigs (>=25000bp) | 224 | 117 |
| Contigs (>=50000bp) | 157 | 104 |
| Total length (>=0bp) | 1085818362 | 586922753 |
| Total length (>=1000bp) | 1085818362 | 586922753 |
| Total length (>=5000bp) | 1085818362 | 586922753 |
| Total length (>=10000bp) | 1085810895 | 586912877 |
| Total length (>=25000bp) | 1085519987 | 586714668 |
| Total length (>=50000bp) | 1083073763 | 586241674 |
| contigs | 239 | 130 |
| Largest contig | 49656299 | 43145923 |
| Total length | 1085818362 | 586922753 |
| GC (%) | 33.02 | 34.69 |
| N50 | 33230923 | 20543509 |
| L50 | 14 | 10 |
| N's per 100 kbp | 0.00 | 0.00 |

**Supplementary Tables S6** Repeat classes of genome assemblies of *L. insolitus* and *P. brunnea*

| Repeats (%) | *L. insolitus* | *P. brunnea* |
| --- | --- | --- |
| **Retroelements** | 31.42 | 13.75 |
| SINEs | 3.89 | 5.1 |
| Penelope | 4.81 | 3.39 |
| LINEs | 27.31 | 8.52 |
| LTR | 0.22 | 0.14 |
| **DNA transposons** | 9.81 | 2.66 |
| **Rolling circles** | 1.22 | 0.08 |
| **Unclassified** | 18.05 | 22.64 |
| **Total interspersed repeats** | 59.28 | 39.06 |
| **Small RNA** | 3.37 | 4.93 |
| **Satellites** | 0.01 | 0 |
| **Simple repeats** | 1.19 | 0.59 |
| **Low complexity** | 0.11 | 0.09 |

**Supplementary Table S7:** Annotation statistics calculated with agat_sp_statistics.pl

|  | *L. insolitus* | *P. brunnea* |
| --- | --- | --- |
| Number of genes | 16306 | 17621 |
| Number of mrnas | 16306 | 17621 |
| Number of mrnas with utr both sides | 16295 | 17614 |
| Number of mrnas with at least one utr | 16306 | 17621 |
| Number of cdss | 16306 | 17621 |
| Number of exons | 127497 | 136127 |
| Number of five_prime_utrs | 16306 | 17621 |
| Number of three_prime_utrs | 16295 | 17621 |
| Number of exon in cds | 117302 | 124905 |
| Number of exon in five_prime_utr | 23390 | 25586 |
| Number of exon in three_prime_utr | 18404 | 20131 |
| Number of intron in cds | 100996 | 107284 |
| Number of intron in exon | 111191 | 118506 |
| Number of intron in five_prime_utr | 7084 | 7965 |
| Number of intron in three_prime_utr | 2109 | 2517 |
| Number gene overlapping | 164 | 389 |
| Number of single exon gene | 1558 | 1473 |
| Number of single exon mrna | 1558 | 1473 |
| mean mrnas per gene | 1 | 1 |
| mean cdss per mrna | 1 | 1 |
| mean exons per mrna | 7.8 | 7.7 |
| mean five_prime_utrs per mrna | 1 | 1 |
| mean three_prime_utrs per mrna | 1 | 1 |
| mean exons per cds | 7.2 | 7.1 |
| mean exons per five_prime_utr | 1.4 | 1.5 |
| mean exons per three_prime_utr | 1.1 | 1.1 |
| mean introns in cdss per mrna | 6.2 | 6.1 |
| mean introns in exons per mrna | 6.8 | 6.7 |
| mean introns in five_prime_utrs per mrna | 0.4 | 0.5 |
| mean introns in three_prime_utrs per mrna | 0.1 | 0.1 |
| Total gene length | 456694588 | 284782984 |
| Total mrna length | 456694588 | 284782984 |
| Total cds length | 23836285 | 24644446 |
| Total exon length | 32299475 | 36705243 |
| Total five_prime_utr length | 2231123 | 2789468 |
| Total three_prime_utr length | 6232067 | 9271329 |
| Total intron length per cds | 344892006 | 194671693 |
| Total intron length per exon | 424395113 | 248077741 |
| Total intron length per five_prime_utr | 58313895 | 43283296 |
| Total intron length per three_prime_utr | 5827154 | 4332670 |
| mean gene length | 28007 | 16161 |
| mean mrna length | 28007 | 16161 |
| mean cds length | 1461 | 1398 |
| mean exon length | 253 | 269 |
| mean five_prime_utr length | 136 | 158 |
| mean three_prime_utr length | 382 | 526 |
| mean cds piece length | 203 | 197 |
| mean five_prime_utr piece length | 95 | 109 |
| mean three_prime_utr piece length | 338 | 460 |
| mean intron in cds length | 3414 | 1814 |
| mean intron in exon length | 3816 | 2093 |
| mean intron in five_prime_utr length | 8231 | 5434 |
| mean intron in three_prime_utr length | 2762 | 1721 |
| Longest gene | 1412090 | 718581 |
| Longest mrna | 1412090 | 718581 |
| Longest cds | 87852 | 59568 |
| Longest exon | 32610 | 32277 |
| Longest five_prime_utr | 2249 | 2729 |
| Longest three_prime_utr | 6411 | 7921 |
| Longest cds piece | 32610 | 32277 |
| Longest five_prime_utr piece | 2249 | 2728 |
| Longest three_prime_utr piece | 6411 | 7921 |
| Longest intron into cds part | 862306 | 542153 |
| Longest intron into exon part | 862306 | 542153 |
| Longest intron into five_prime_utr part | 631104 | 478780 |
| Longest intron into three_prime_utr part | 203963 | 145806 |
| Shortest gene | 110 | 109 |
| Shortest mrna | 110 | 109 |
| Shortest cds | 102 | 102 |
| Shortest exon | 1 | 1 |
| Shortest five_prime_utr | 1 | 1 |
| Shortest three_prime_utr | 1 | 1 |
| Shortest cds piece | 1 | 1 |
| Shortest five_prime_utr piece | 1 | 1 |
| Shortest three_prime_utr piece | 1 | 1 |
| Shortest intron into cds part | 30 | 30 |
| Shortest intron intro exon part | 30 | 30 |
| Shortest intron into five_prime_utr part | 50 | 44 |
| Shortest intron into three_prime_utr part | 35 | 45 |

**Supplementary Table S8:** BUSCO statistics of genome annotations with Helixer.

| **BUSCO** | *L. insolitus* | *P. brunnea* |
| --- | --- | --- |
| complete | 87.6% | 92.2% |
| single | 86.4% | 90.9% |
| duplicated | 1.2% | 1.3% |
| fragmented | 5.8% | 5.1% |
| missing | 6.6% | 2.7% |
